# Unintended coastal transformation from small-scale infrastructure and land use change

**DOI:** 10.1038/s41598-025-15377-y

**Published:** 2025-08-22

**Authors:** Warit Charoenlerkthawin, Chaiyut Charoenphon, William C. Burnett, Somboon Otarawanna, Butsawan Bidorn

**Affiliations:** 1 Department of Water Resources Engineering, Chulalongkorn University, Bangkok, 10330 Thailand; 2 Center of Excellence in Interdisciplinary Research for Sustainable Development, Faculty of Engineering, Chulalongkorn University, Bangkok, 10330 Thailand; 3 Department of Survey Engineering, Chulalongkorn University, Bangkok, 10330 Thailand; 4 Department of Earth, Ocean and Atmospheric Science, Florida State University, Tallahassee, FL 32306 USA; 5 National Metal and Materials Technology Center (MTEC), National Science and Technology Development Agency (NSTDA), Pathum Thani, 12120 Thailand

**Keywords:** Coastal structure, Land cover change, LiDAR, Coastal landscape, Coastal management, Civil engineering, Environmental impact, Sustainability, Physical oceanography

## Abstract

This study investigates the impact of small-scale coastal development on beach material changes along the Ban Khlong Wan (BKW) coastline in the Mid-Gulf of Thailand, a site of legal disputes between local communities and government agencies over environmental impacts. We applied shoreline change analysis, high-resolution LiDAR observations, beach material characterization, and land use change assessment to understand the causes of beach transformation. Contrary to prior reports attributing the transition from sandy to muddy conditions to coastal protection structures, our findings reveal the coastline remained predominantly sandy until 2002, with shoreline shifts averaging less than ± 1 m/year. The construction of a fishery pier and hard structures (2004–2008) disrupted longshore sediment transport, leading to sand accumulation updrift and sediment deficits downdrift. This, combined with increased muddy inputs from upstream land use change, particularly the conversion of 100 ha of natural wetland into aquaculture ponds, contributed to mudflat formation. While detached breakwaters and seawalls aided in shoreline stabilization, they were not the primary cause of the transformation. Rather, the shift from alongshore sandy to riverine muddy sediment supply was the key driver. These findings highlight the need for integrated coastal management that considers the interactions between infrastructure, sediment processes, and land use change to mitigate degradation and support sustainable development of small-scale coastal projects.

**Supplementary Information:**

The online version contains supplementary material available at 10.1038/s41598-025-15377-y.

## Introduction

Coastal and marine sustainability is a global challenge, as recognized in the United Nations’ 2030 Agenda for Sustainable Development. Sustainable Development Goal (SDG) 14 emphasizes the conservation and sustainable use of oceans, seas, and marine resources (https://sdgs.un.org/goals/goal14). The ocean plays a critical role beyond SDG 14, supporting economic activities, poverty alleviation, health, and sustainable industrialization^1–3^. However, coastal sustainability is increasingly threatened by sediment supply disruptions, land use changes, and shoreline modifications, contributing to land degradation^3–9^. As nearly 37% of the global population are living in coastal areas, securing sediment balance and shoreline stability is vital for environmental protection and economic sustainability^10^. However, coastal zones are facing growing pressures from infrastructure expansion, hydrodynamic alterations, and upstream land use modifications^11–13^.

Fisheries play a crucial role in sustaining the economies of coastal communities, providing essential livelihood and employment^14,15^. Strengthening the fisheries sector, especially by investing in better infrastructure, can directly benefit local communities and help advance global food security, while also contributing to several Sustainable Development Goals^16^. However, infrastructure developments such as fishery ports and breakwaters, while intended to improve coastal access and reduce erosion, often come with unintended side effects. These can include loss of coastal habitats, changes to natural sediment transport, and long-term morphological changes to the shoreline^17,18^.

One proposed approach to managing these impacts is the “EcoFishing Port” concept, which promotes more environmentally responsible operations at fishery ports^19^. Generally, Environmental Impact Assessments (EIAs) are the primary tools for evaluating the impacts of such projects, by measuring physical and biological parameters such as waves, currents, tides, sediment dynamics, and water quality^6,20–23^. However, these assessments often place limited emphasis on the cumulative or long-term socio-economic consequences of infrastructure development, especially in regions where post-construction monitoring is minimal. In such contexts, the lack of follow-up evaluation has triggered environmental degradation or conflict among stakeholders^24–26^.

**Fig. 1:**
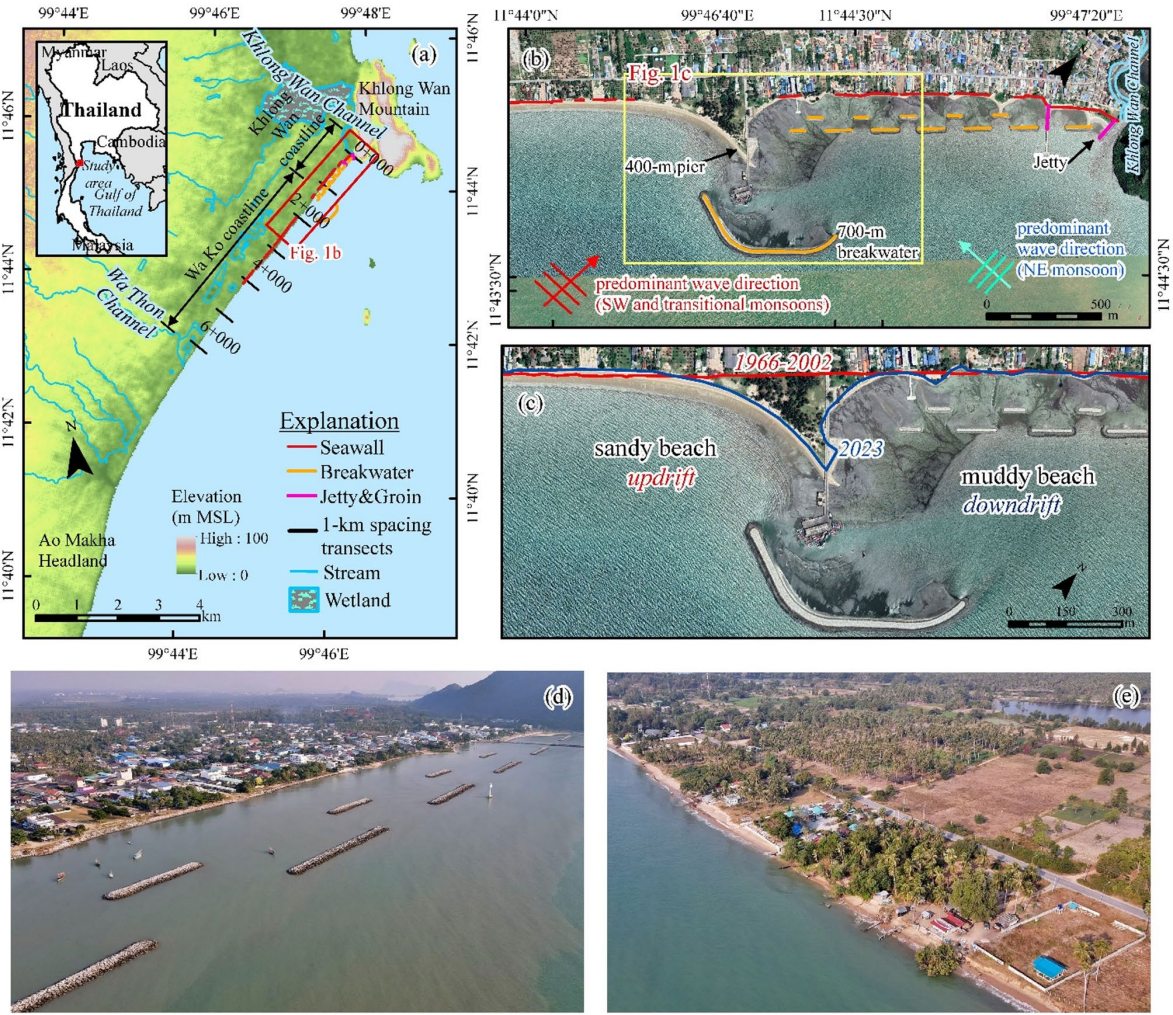
Study area: (**a**) Shoreline change measurement transects spaced 1 km apart from Khlong Wan Channel to Wa Thon Channel. (**b**) Coastal structures within the study area. (**c**) Shoreline changes before and after coastal development, with updrift beaches characterized by sandy textures and downdrift areas predominantly muddy. (**d**) The Khlong Wan coastline, protected by seawalls, serves as a major community and tourism area. (**e**) The updrift coastline features agricultural lands and small businesses. The maps were generated using ArcMap software version 10.6 (ESRI Inc.). The satellite images of b and c were downloaded from Google Earth Pro (©Maxar Technologies, https://www.google.com/earth). The images d–e were taken by author.

Similar issues have been observed worldwide, especially where coastal development for tourism or small-scale fisheries have led to problems such as shoreline erosion, habitat destruction, and disputes over land rights^18,27–29^. In Thailand, recent fishery port development along the Ban Khlong Wan (BKW) coastline, including the construction of a 400-meter fishery pier and a series of coastal protection structures (Fig. 1a–e), have altered sediment transport dynamics and significantly impacted coastal environment. Coastal degradation after the project construction resulted in lawsuit between local communities and government agencies^30^ Although the community, stakeholders, and NGOs have attributed these negative impacts to seawalls and breakwaters, a clear understanding of the causes is limited. Without a comprehensive understanding of the interaction among infrastructure development, hydrodynamic shifts, and upstream land use changes have contributed to ongoing coastal degradation. This knowledge gap has been a key factor in a legal dispute in Ban Khlong Wan that has continued for more than a decade (https://beachforlife.org/blog/37).

The objectives of this study are to investigate the role of small-scale fishery infrastructure in sediment dynamics and coastal transformation, emphasizing the interactions between coastal structures, sediment supply shifts, and land use changes. We integrate shoreline change analysis, LiDAR-based monitoring, beach material characterization, and land use change assessments to: (1) quantify long-term coastal changes in response to fishery infrastructure expansion and hydrodynamic alterations; (2) identify key drivers of sediment disruption, particularly changes in alongshore transport and riverine sediment supply; and (3) develop science-based strategies for sustainable coastal management to minimize unintended environmental degradation from small-scale fishery projects. Our integrated geospatial and sedimentological approach and policy-relevant outcomes can inform sustainable planning and impact assessments for small- to medium-scale coastal infrastructure worldwide, particularly in rapidly developing or ecologically sensitive coastal zones.

## Study area

The BKW coast, situated in the Mid-Gulf of Thailand, extends approximately 12 km from Khlong Wan Hill to Ao Makha headland, spanning 11°43’ to 11°45’ N latitude and 99°46’30” to 99°47’30” E longitude (Fig. 1a). The climate of the study area is influenced by the southwest (SW) monsoon from May to October and the northeast (NE) monsoon from November to January, significantly affecting temperature, rainfall, and wave conditions^31,32^. The SW monsoon typically results in calmer wave conditions with heights generally below 0.5 m, while the NE monsoon brings larger waves generally ranging from 0.5 to 1.0 m from the NNE. Stronger wave conditions (heights up to 1.5 m) also occur during the transitional period (October–February), primarily originating from the south^33,34^. The BKW coast is considered an intertidal zone with average tidal ranges of 1.0 m during neap tides and 1.3 m during spring tides^33^. The beach materials along BKW mainly consist of sand, silt, and mud^32^. The BKW littoral zone receives sandy longshore sediment from the south, while the Khlong Wan channel, located at the northernmost part of the coastline (Fig. 1a), supplies muddy sediment and organic matter, especially during the wet season when heavy rainfall increases channel discharge^33^.

Various types of fisheries are an important part of the local economy for villagers living along this coast. Since the Khlong Wan channel is not a permanent channel, vessel traffic depends upon tides. In 2007 and 2008, a 400-m long pier and a 700-m long offshore breakwater were built 1.5 km south of the Khlong Wan channel to support the local fisheries (Fig. 1b). To mitigate shoreline erosion caused by downdrift effects of the pier construction, twelve detached breakwaters were implemented between the Khlong Wan channel and the fishery pier, an area of significant value for the local economy (Figures. 1b–d). To the north of the pier, the land is primarily used for residential areas, hotels, and a public park, which is mainly protected by seawalls (Fig. 1d), while the land to the south is predominantly used for agriculture (Fig. 1e). Following the construction of these coastal structures, the BKW coastal system became divided into two sub-systems: the Khlong Wan and Wa Ko coastlines (Fig. 1a). This division triggered significant coastal environmental transformations, particularly along the Wa Ko coastline, where the sandy beach gradually transitioned into a muddy coast (Figs. 1b–c and S1). The unintended beach transformation of these structural interventions exacerbated coastal degradation, causing undesirable beach transformation and shoreline changes. These environmental impacts ignited conflicts between residents and government agencies, ultimately resulting in legal disputes filed by local communities against the authorities^30^. The plaintiffs called for the removal of the structures and demanded a USD $300,000 environmental restoration fund. Although the Central Administrative Court acknowledged procedural flaws, it ruled that the projects did not breach EIA laws, since construction occurred before the relevant regulations came into force. The decision highlighted governance shortcomings but did not mandate remediation. This case underscores the importance of understanding the geomorphic and ecological consequences of small-scale coastal infrastructure and calls for evidence-based guidance to inform more sustainable coastal development practices.

## Methods

To establish a comprehensive understanding of coastal changes at BKW, our study integrates multiple lines of evidence. Long-term shoreline change analysis, derived from satellite imagery and historical aerial photographs, was conducted to quantify pre- and post-construction shoreline dynamics. UAV-based LiDAR data provided high-resolution topographic information, enabling precise assessment of vertical and horizontal morphological changes. Sediment sampling was employed to validate the presence and distribution of fine-grained material along the beach, supporting inferences drawn from remote sensing data. Additionally, land use analysis within the upstream catchment was undertaken to evaluate potential anthropogenic influences on sediment supply. By synthesizing shoreline position data, digital terrain models, sediment properties, and land use changes, we constructed a cohesive framework to investigate the cumulative impacts of coastal infrastructure on shoreline evolution and sediment redistribution. The details of each method are provided in the subsections below.

### Shoreline change analysis

To assess the impacts of coastal development on the BKW coastline, historical shoreline positions along a 6.5 km stretch from Khlong Wan Channel to Wa Thon Channel (Fig. 1a) were analyzed from 1966 to 2023. Shoreline data were derived from aerial photographs (1966 and 1976) with scale of 1:50,000, an orthophotograph (2002) with a scale of 1:4,000 provided by the Royal Thai Survey Department (RTSD), and satellite imagery (2013–2023) with a spatial resolution of 0.5–0.8 m obtained via Maxar Technologies, CNES, and Airbus through Google Earth Pro software (version 7.3) as shown in Table S1. All images were georeferenced using ArcGIS version 10.6 (Environmental Systems Research Institute, Inc., California, U.S.), an accepted protocol for shoreline change studies^23,35–37^.

Shoreline proxies, such as the edge of vegetation, roads, and dikes were used to delineate the shoreline positions at high-water-line^38^ for seven time periods (1966–2023). The shoreline changes before and after coastal development projects were evaluated using the Digital Shoreline Analysis System (DSAS) version 5.1. A total of 261 transects were generated at 25 m intervals, perpendicular to a baseline from Khlong Wan Channel (0 + 000) to Wa Thon Channel (6 + 500) as illustrated in Fig. 1a. Transect numbers like “6 + 000” indicate distances in meters from the baseline, with the first number representing kilometers and the second representing additional meters. The Net Shoreline Movement (NSM) and End Point Rate (EPR) models^39^ were used to calculate the displacement and rate of shoreline change for each transect. This methodological system is known for its global application in shoreline change studies and is particularly effective for analyzing the effects of coastal structures^37–42^.

Shoreline position uncertainties were primarily associated with pixel errors from the imagery and rectification processes^43–45^with an uncertainty of less than 1 m for satellite images and less than 3 m for aerial photographs. These levels of uncertainty are considered acceptable for studying the impacts of coastal structures on shoreline dynamics^13,46,47^. Further details on the general methodology and error estimations can be found in the Supplementary Materials.

### Beach materials sampling and analysis

To investigate beach transformation, sediment samples were collected from 36 locations along a 6 km stretch of the BKW coastline, specifically between Khlong Wan Channel and Wa Thon Channel (Fig. 1a), to capture a broad representation of updrift and downdrift sediments near the pier and coastal protection structures. The samples, generally collected from the top 15 cm of the bed surface, underwent grain size analysis using standard methods. Dry sieving analysis was conducted for coarse, non-cohesive materials, while wet sieving analysis was applied for samples containing a mixture of cohesive and non-cohesive materials. For finer particles, such as silt and clay, hydrometer analysis was performed. All procedures followed ASTM standards^48^which are detailed in Supplementary Materials.

Beach materials were analyzed through grain size distribution to classify sediment types and identify spatial variations in sediment composition. Particular attention was given to comparing the relative proportions of fine-grained materials (e.g., clay and silt) between the updrift and downdrift zones of the study area. This comparison was designed to detect the presence of fine sediment input following the construction of coastal infrastructure. Given that sediment contributions from the adjacent channel were previously considered negligible in earlier assessments, this analysis aimed to reassess that assumption. The results provide supporting evidence for evaluating the influence of small-scale fluvial inputs on coastal sedimentation under varying hydrodynamic conditions.

### LiDAR observations and topography

We conducted LiDAR observations on January 23, 2023 along a 3-km segment spanning Khlong Wan and Wa Ko coastlines to evaluate the impact of coastal structures on sediment transport along the updrift and downdrift sections of the coastline. Beach topography data were collected using a DJI Matrice 300 RTK drone equipped with a Zenmuse L1 LiDAR sensor, known for its high precision and resolution^3^,49^50^, . This LiDAR survey provided detailed topographical mapping with a dense point cloud^51^featuring approximately 5 cm spacing between points.

To ensure the accuracy of the data, ground truthing was performed using a STONEX S10 GNSS device, and Real Time Kinematic (RTK) methods were employed to validate the UAV-LiDAR data, achieving positional uncertainties within a few centimeters, as supported by previous studies^52–55^. The Root Mean Square Error (RMSE) of the LiDAR-derived elevation data, calculated by comparing it with RTK survey measurements, was determined to be only 3 cm. Further details on the LiDAR survey setup, ground truthing methods, and RMSE calculations are provided in Supplementary Materials.

We compared two datasets: the 1997 DTM, derived from 1-m interval hydrographic contour data surveyed by the Harbour Department^33^ (Figure S2a), and the 2023 high-resolution DTM obtained from our UAV-LiDAR survey (Figure S2b) to estimate the impact of the fishery pier, offshore and detached breakwaters, and seawalls on nearshore morphology of BKW coastline. The sediment volume above − 1 m MSL trapped updrift and downdrift following its construction was estimated and compared with results from a numerical model (Harbour Department^33^. The model, which relied on inland wind data to estimate wave characteristics and sediment transport, provided a theoretical framework for understanding sediment dynamics. Given the inherent complexity of coastal processes, our approach can assess the uncertainties in coastal morphological changes associated with using synthesized wave characteristics derived from wind data. This underscores the importance of observational data in accurately evaluating the environmental impacts of coastal projects.

### Land use change analysis

Beach sediment characteristics are primarily influenced by sediment sources within the littoral zone, with land use in upstream basins playing an important role in determining sediment grain size^54^. We examined land use changes in a 180-ha wetland area, located approximately 3 km upstream from the Khlong Wan channel, the primary riverine sediment source for the Khlong Wan littoral zone. Given the significance of riverine sediment loads in coastal environments, the conversion of wetlands to aquacultural or agricultural uses can alter soil composition and affect sediment types discharged into the sea. Since such impacts are often overlooked in small-scale coastal project studies including the Khlong Wan development project study, we analyzed historical land use by manually digitizing aerial photographs and satellite images from 1966 to 2023, enabling precise delineation of land use types^57,58^. Three main land use categories were identified: natural wetlands, aquaculture, and agriculture. Wetlands, located in the intertidal zone, are subject to tidal flooding and serve as unique ecological habitats, whereas aquacultural and agricultural areas are enclosed by man-made dikes (Figure S3), facilitating their classification in remote sensing imagery.

## Results

### Shoreline changes pre- and post-coastal development

**Fig. 2:**
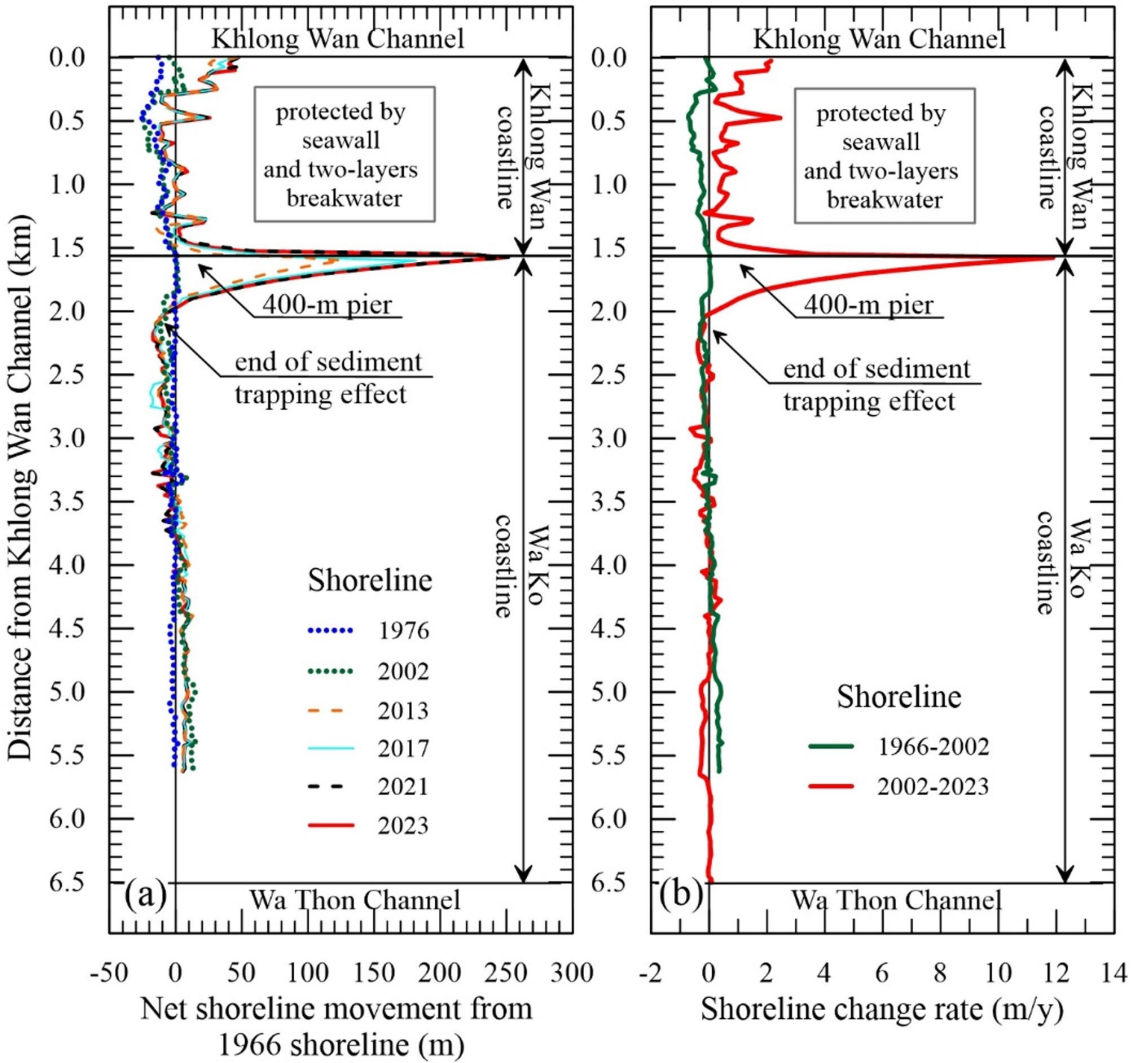
Shoreline change results: (**a**) Net shoreline movement for different periods relative to the 1966 shoreline. (**b**) Shoreline change rates before construction (1966–2002) and after construction (2002–2023).

The historical shoreline changes along the BKW coastline from 1966 to 2023 are presented in Fig. 2, with evaluation periods divided into pre-development (1966–2002) and post-development (2002–2023) phases to assess the impact of coastal structures. The key developments, including a 400-m fishery pier, a 700-m breakwater, two-layer of 12 detached breakwaters constructed by the Habour Department in 2007–2008, and a seawall constructed by Khlong Wan Municipality in 2005 for a public park. Due to the unavailability of shoreline data during this period, the 2002 shoreline, representing a stable condition, was used as the baseline for comparison with the earliest post-construction data from 2013. Table 1 presents the results of the shoreline analysis along the BKW coastline, which is divided into two sections: the Khlong Wan coastline (1.5 km) in the north and the Wa Ko coastline (5 km) in the south. Positive values in Table 1 denote shoreline advance, while negative values indicate shoreline retreat.

**Table 1:** Shoreline change results in pre- and post-construction of coastal development project.

Parameters	Pre-construction (1966–2002)		Post-construction (2002–2023)	
	Khlong Wan coastline	Wa Ko coastline	Khlong Wan coastline	Wa Ko coastline
STA. (number of transects)	0 + 000 to 1 + 550 (63)	1 + 575 to 6 + 500 (198)	0 + 000 to 1 + 550 (63)	1 + 575 to 6 + 500 (198)
Mean NSM (m)	–10	1	18	6
Minimum NSM (m)	–26	–12	–3	–13
Maximum NSM (m)	8	16	77	250
Accretion (%)	8.0	33.3	96.8	33.3
Retreat (%)	82.5	27.3	1.6	51.5
Stable (%)	9.5	39.4	1.6	15.2
Uncertainty of NSM (m)	± 3	± 3	± 0.8	± 0.8
Shoreline change rates using EPR (m/y)				
Mean shoreline change rate	–0.28	0.04	0.84	0.28
Maximum accretion rate	0.23	0.45	3.67	11.89
Mean accretion rate	0.15	0.24	0.86	1.17
Maximum retreat rate	–0.72	–0.32	–0.16	–0.64
Mean retreat rate	–0.36	–0.19	–0.16	–0.22
Uncertainty of EPR	± 0.08	± 0.08	± 0.05	± 0.05

Note: NSM = net shoreline movement; EPR = end point rate.

Before the coastal development project, the Khlong Wan coastline experienced shoreline retreat, with 83% of the area eroding at an average rate of − 0.3 ± 0.08 m/y, particularly between 0.25 and 1.3 km from the Khlong Wan Channel (Figs. 2a–b). In contrast, the Wa Ko coastline showed greater stability and accretion, with 39.4% remaining stable and 33.3% accreting, especially near the Wa Thon Channel (Fig. 1a), where the accretion rate averaged 0.24 ± 0.08 m/y. Overall, the average shoreline change rate along both coastlines was less than ± 0.5 m/y, indicating a generally stable coastline before the coastal development.

Following the construction of the 400-m pier, the Wa Ko coastline experienced significant changes. The most notable alteration occurred within 400 m south of the pier, where substantial accretion was observed. The shoreline advanced by up to 250 m, corresponding to an accretion rate of approximately 12 m/y. The land accretion of approximately 40,000 m^2^ was primarily caused by the interruption of longshore sediment transport by the pier, which acted as a sediment trap, confirming the dominant south-to-north sediment movement along this stretch of coastline. The shoreline position data also reveal that accretion due to the pier has ceased extending seaward since 2021 (Fig. 2a), indicating that the sediment trapping capacity of the pier may have reached an equilibrium after about 13 years.

Along the Khlong Wan coastline, the construction of coastal defense structures, including two-layer detached breakwaters and seawalls, (Fig. 1b and d) significantly contributed to the stabilization and seaward advancement of the shoreline. After construction, the average NSM for the entire Khlong coastline was 18 m, with shoreline change rates ranging from − 0.2 to 3.7 m/y. As shown in Table 1 and 97% of the Khlong Wan coastline experienced accretion, largely due to the addition of 90,500 m³ of sand by the Harbor Department as part of erosion control efforts. The area within 50 m north of the pier also experienced significant accretion, with shoreline advances ranging from 6 to 77 m.

### Beach material characterization

**Fig. 3:**
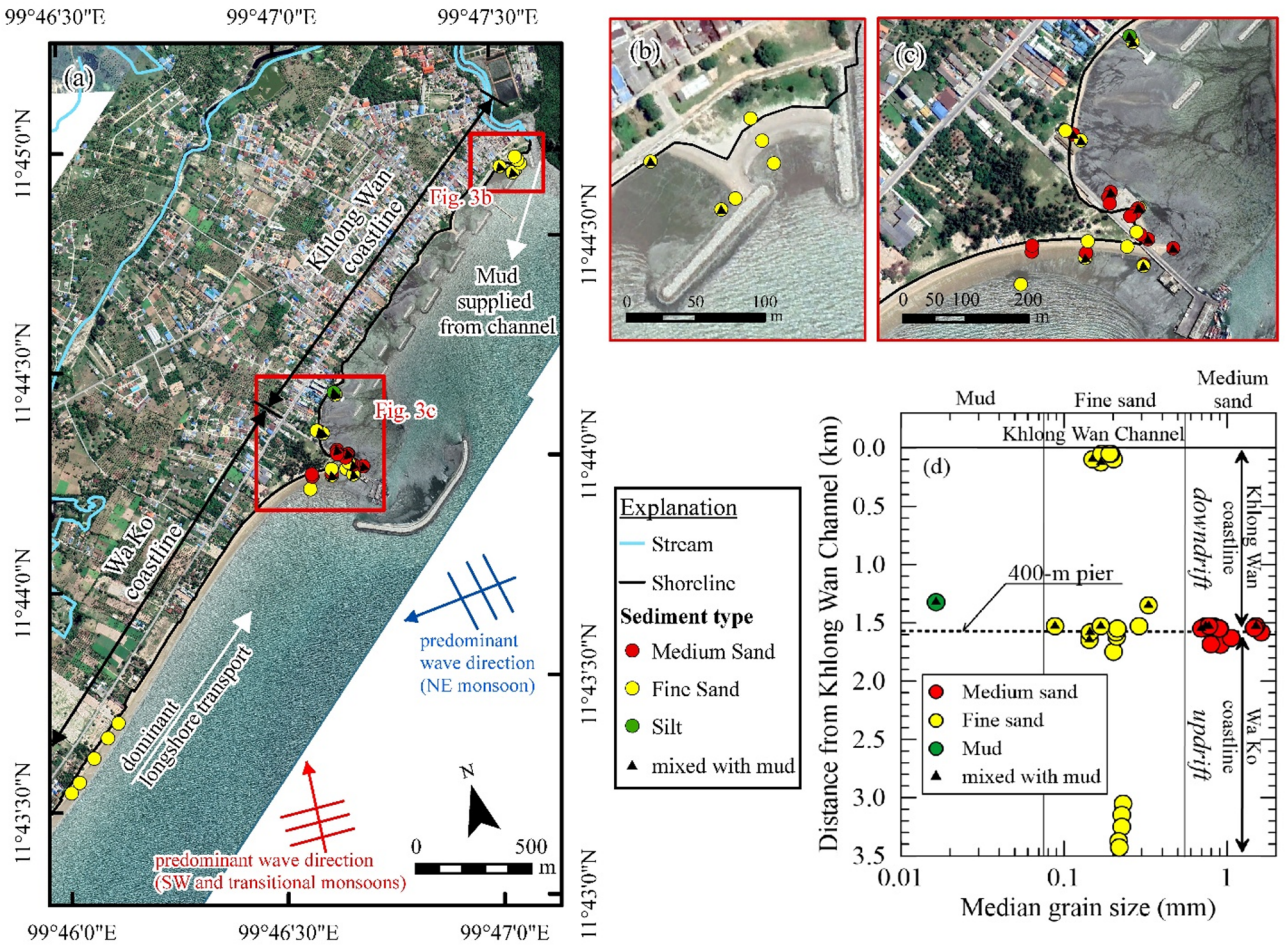
Results of beach material sampling and analysis. (**a**) Sampling locations and classification of each sample. (**b**) Detailed view of sampling locations near the Khlong Wan Channel. (**c**) Detailed view of sampling locations near the 400-m pier. (**d**) Grain size distribution along the downdrift and updrift coastline of the pier. The maps were generated using ArcMap software version 10.6 (ESRI Inc.). The satellite images of a and c were downloaded from Google Earth Pro (©Maxar Technologies, https://www.google.com/earth).

On January 23, 2023, 36 beach material samples were collected along a 3.5 km stretch of the study area, divided into updrift (Wa Ko) and downdrift (Khlong Wan) coastlines relative to the fishery pier (Figs. 3a–c). Updrift samples from 14 locations had median grain sizes ranging from 0.14 mm to 1.61 mm (average 0.44 mm), with 71% classified as fine sand and 29% as medium sand, primarily near the pier. Minimal mud and shell fragments were observed, mostly in samples below mean sea level (Figure S1a). Downdrift, 22 samples showed median grain sizes between 0.016 mm and 1.51 mm (average 0.58 mm), with 95% composed mainly of sand, while 50% contained 3–91% mud. One sample, collected behind a detached breakwater near the pier, was classified as mud (0.016 mm), while six samples near the Khlong Wan channel contained fine sand with 23–29% mud. Field observations confirmed that mud mixtures were predominantly found below mean sea level along the Khlong Wan coast (Figure S1b). The results are summarized in Table S2 and visualized in Fig. 3d.

### Evolution of beach topography post-coastal development

**Fig. 4:**
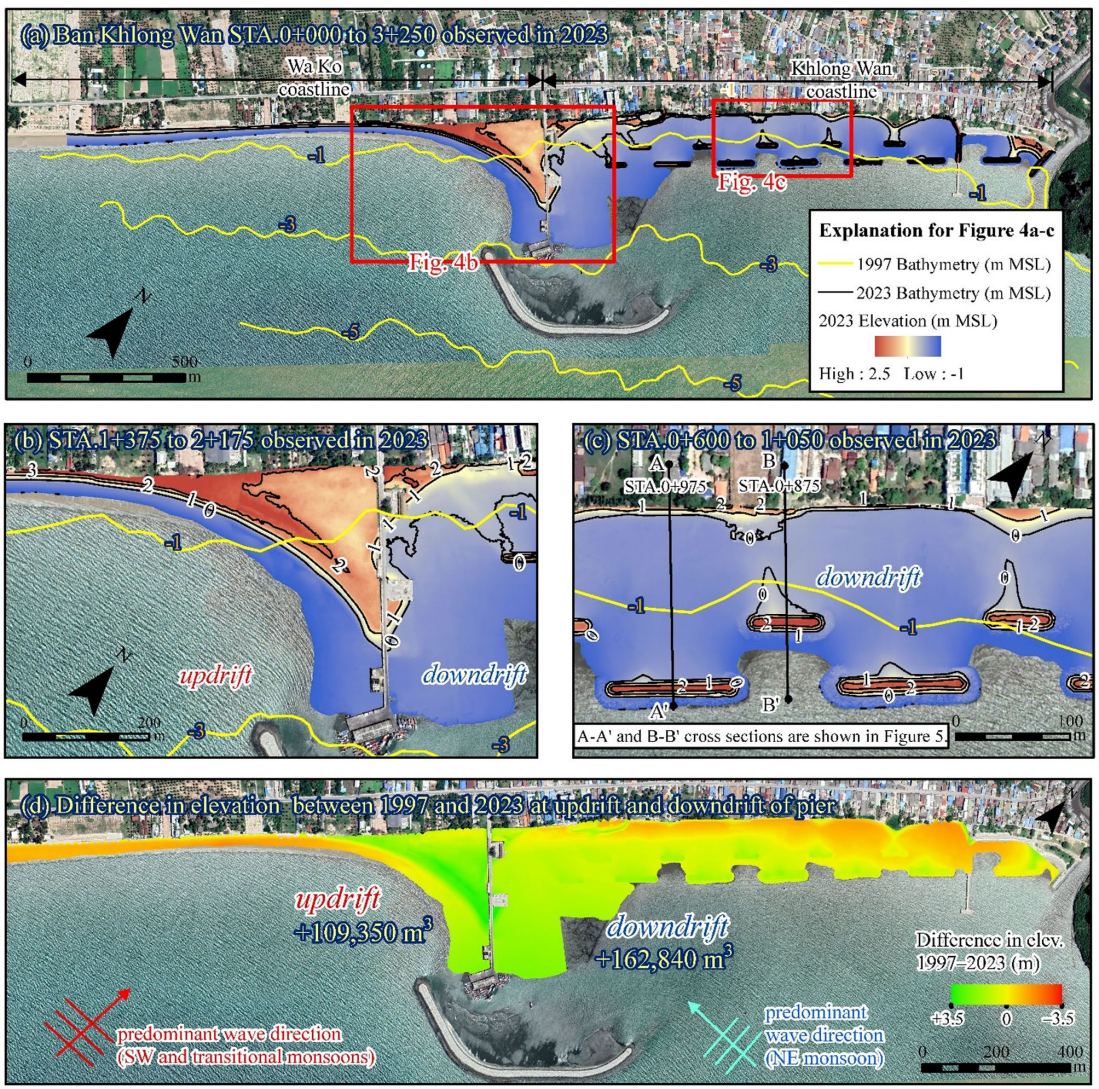
LiDAR observation results: (**a**) digital terrain model generated from LiDAR point cloud data covering the Khlong Wan and Wa Ko coastlines. (**b**) Detailed view showing higher beach elevations south of the pier (updrift) compared to lower elevations north of the pier (downdrift). (**c**) Topographic details of the downdrift coastline, highlighting mudflat elevations and seabed responses to inner and outer detached breakwaters. (**d**) Elevation changes observed between 1997 and 2023. The maps were generated using ArcMap software version 10.6 (ESRI Inc.). The satellite images of a–d were downloaded from Google Earth Pro (©Maxar Technologies, https://www.google.com/earth).

The beach topography along a 3.1-km coastline, observed via UAV-LiDAR, is illustrated in Fig. 4a–c. The comparison between the 1997 bathymetric data with our UAV-LiDAR data for the area above − 1 m MSL in 2023 reveals the changes in nearshore sediment volume pre- and post-construction along the updrift and downdrift coastlines, as shown in Table 2; Fig. 4d. Based on Fig. 4d, substantial seaward expansion of the sandy beach was observed south of the pier (Wa Ko coastline), covering an accretion area of 45,000 m^2^. Sediment deposits reached up to 3.5 m thick, contributing to nearshore sediment deposition of 109,000 m³ during 1997–2023. Additionally, significant beach loss was found near the shore at the southernmost part of the Wa Ko coast, where the seabed elevation decreased by up to − 1.8 m. On the downdrift side (Khlong Wan coastline), sand accumulation was observed adjacent to the pier, but at a considerably lower rate, covering an accretion area of only 4,000 m^2^.

**Table 2:** Summary of sediment volume analysis for the Updrift (Wa Ko coastline) and downdrift (Khlong Wan coastline) between 1997 and 2023.

Parameters	Updrift (Wa Ko coastline)	Downdrift (Khlong Wan coastline)
Sand accretion area above MSL (m^2^)	45,000	4,000
Sand deposition around the pier above MSL (m^3^)	81,000	9,000
Total deposition volume above − 1 m MSL (m^3^)	109,000	163,000
Total erosion volume above − 1 m MSL (m^3^)	–39,000	–45,000
Difference in seabed elevation (m) [average]	–1.8 to 3.5 [0.65]	–1.7 to 3.5 [0.50]

**Fig. 5:**
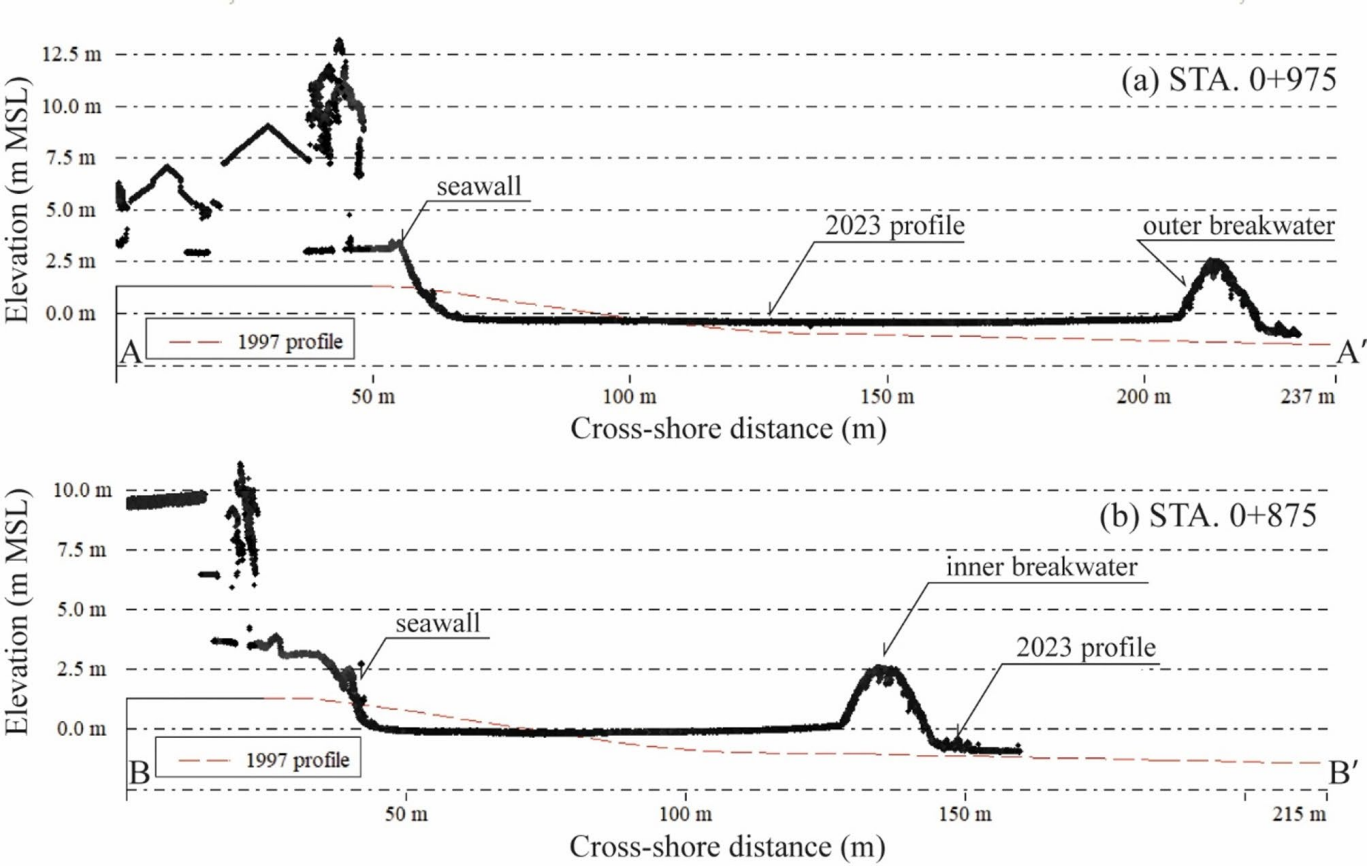
Seabed response to breakwater construction downdrift of the pier: (**a**) outer breakwater at STA. 0 + 975, and (**b**) inner breakwater at STA. 0 + 875. The red-dashed line shows the 1997 profile, and the black points represent 2023 LiDAR observations. These profiles confirm the breakwaters’ roles in dissipating wave energy and promoting sediment deposition. Cross-section locations (A-Aʹ and B-Bʹ) are shown in Fig. 4c.

However, significant mud deposition was identified along the Khlong Wan coastline, with intense accumulation near the pier, where maximum deposit thickness reached up to 2.0 m. The deposition depth gradually decreased toward the north and the mud accumulation was mainly found behind the detached breakwaters with the deposition depth of 0.5–1.0 m. This process resulted in a nearshore sediment accumulation of approximately 163,000 m³ compared to the pre-construction. Despite this deposition, beach erosion was observed in front of the seawalls along the Khlong Wan coastline (Figs. 4b and 5), leading to sediment loss near the shore of about − 45,000 m³ during this period.

### Land use changes upstream of the coastal zone

**Fig. 6:**
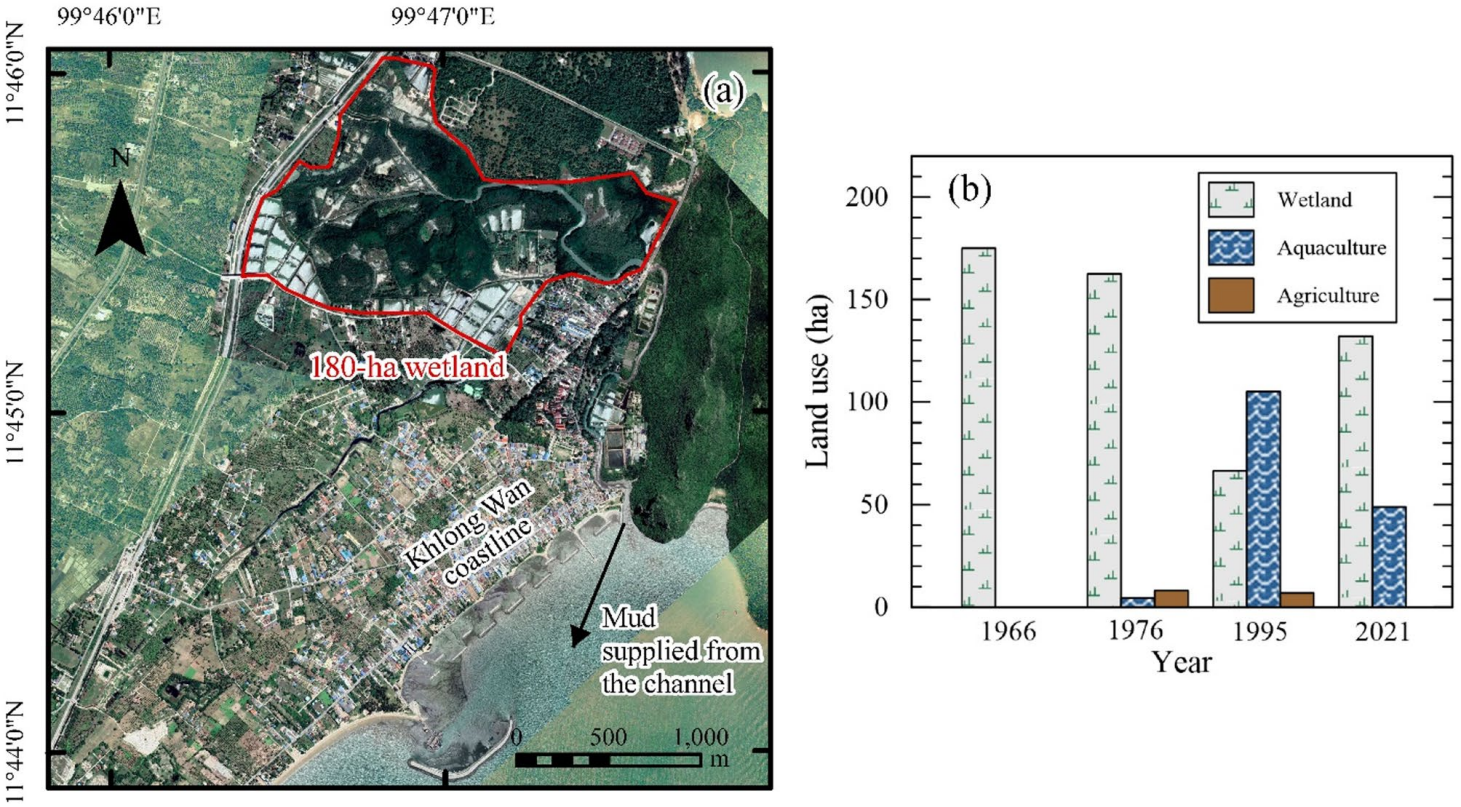
Land use changes upstream of the Khlong Wan Channel. (**a**) Location of the wet land situated 3 km upstream from the BKW coastline. (**b**) Land use changes observed between 1966 and 2021. The map was generated using ArcMap software version 10.6 (ESRI Inc.). The satellite image of panel (**a**) was downloaded from Google Earth Pro (©Maxar Technologies, https://www.google.com/earth).

The Khlong Wan Channel serves as a sediment source for the Khlong Wan littoral zone (Fig. 1a), though its contribution has been considered minimal due to its small watershed and short channel length. However, as the only source of fine sediment supply to the coast, changes in land use within its basin can directly influence sediment dynamics in the coastal zone. Figure 6a illustrates the location of the wetland and provides data on land use changes from 1966 to 2021 (Fig. 6b), along with a series of images showing the evolution of land use across four different periods (Figures S3a–d).

Between 1966 and 1976, the area was predominantly covered by natural wetlands, spanning over 180 ha (Figure S3a). By 1995, 58% of these wetlands (100 ha) had been illegally converted into aquaculture farms, driven largely by the promotion of aquaculture in Thailand during that period (Fig. 6b). This transformation involved extensive landscape modifications, including land occupation, dike construction, vegetation clearance, and soil excavation, as shown in Figures S3b–c. In response to these changes, the Royal Forest Department initiated interventions after 2002 to reclaim illegally encroached land, leading to the reforestation of approximately 50% of the former aquaculture areas with mangroves (Figure S3d). As a result, by 2021, the aquaculture area had been reduced to only 50 ha.

The integration of shoreline change analysis, sediment sampling, UAV-LiDAR topographic monitoring, and upstream land use assessment provides a comprehensive understanding of how small-scale coastal infrastructure has altered sediment dynamics and morphology along the BKW coastline. Post-construction shoreline monitoring reveals that the 400-m fishery pier significantly obstructed longshore sediment transport, leading to pronounced accretion on the updrift Wa Ko side, while downdrift areas showed mixed responses of localized accretion and erosion. Sediment sampling further confirmed the unexpected presence of fine-grained material (clay and silt) on the downdrift Khlong Wan coast, likely sourced from the nearby channel, contradicting earlier assumptions that its sediment contribution was negligible. UAV-LiDAR data exhibited obvious elevation gains behind detached breakwaters, indicating effective sediment trapping, while erosion persisted in front of seawalls. These coastal changes are further linked to historical land use transformations upstream, where wetland-to-aquaculture conversion in the Khlong Wan basin likely intensified fine sediment transport to the coast.

## Discussion

### Coastal sediment processes along the ban Khlong Wan coastline

Shoreline evolution along the (BKW coast is governed by complex interactions between sediment sources and transport mechanisms. Two key sources contribute to sediment dynamics in this area are sandy littoral drift driven by wave action and muddy sediment delivered by the Khlong Wan channel. In the original feasibility study for the fishery pier and breakwater, the Harbour Department estimated net northward sediment transport of approximately 72,800 m³/year during the southwest and transitional monsoon seasons, which was nearly triple the southward transport predicted for the northeast monsoon. These estimates were based on theoretical modeling using inland wind data and considered only sandy sediment. Fine sediment from the Khlong Wan channel was excluded as the channel’s small watershed was assumed to be insignificant in terms of sediment supply.

**Fig. 7:**
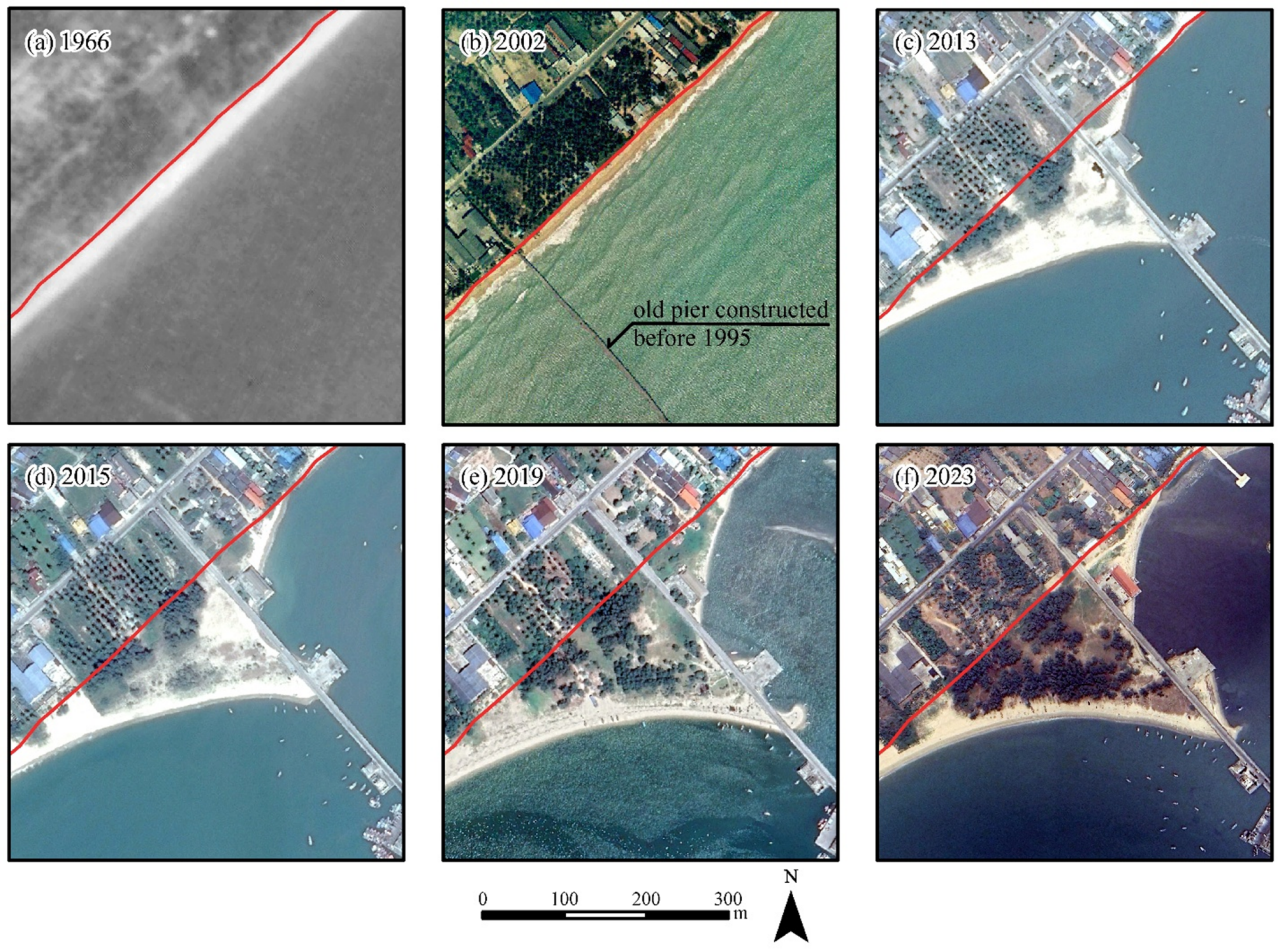
Shoreline evolution during 1966–2023. The red line represents the 1966 shoreline. The maps were generated using ArcMap software version 10.6. Aerial photograph and orthophotograph of a and b were obtained from Royal Thai Survey Department. The satellite images of c–f were downloaded from Google Earth Pro (©Maxar Technologies, https://www.google.com/earth).

However, our post-construction field observations and historical shoreline analyses reveal notable discrepancies from these initial projections. Historical images (Fig. 7) confirm sandy accretion updrift of the 400-m pier (Figs. 7c–f), which generally aligns with model predictions confirming nearshore sand transport. However, substantial muddy sediment deposits were also observed downdrift of the pier and behind the 700-m-long breakwater (Fig. 1b). These deposits suggest the presence of a secondary transport pathway dominated by fine sediment, which is likely to move offshore in suspension (Figure S4) and settling in calmer zones. This dynamic was not captured by the original numerical simulation, which lacked representation of suspended sediment processes in deeper waters.

Our findings also highlight a key oversight in the initial environmental assessment, especially underestimation of the geomorphic role of fine sediments. Although the Khlong Wan channel was excluded from the sediment budget due to its limited catchment area, the field evidence indicates that muddy sediment from this source contributed significantly to post-construction sedimentation patterns. This is particularly evident in the spatial separation between sandy accretion updrift and muddy deposition downdrift, revealing the different transport behaviors of coarse and fine particles under various hydrodynamic conditions. These observations suggest that even minor fluvial inputs can become geomorphologically important when interacting with built structures that alter local flow regimes.

Additionally, the pier’s obstruction of littoral drift appears to modify local hydrodynamics creating conditions favorable for fine sediment entrapment behind the breakwaters. The interaction between engineered structures and previously disregarded sediment sources emphasizes the need to reevaluate assumptions about source significance in coastal modeling, especially in areas with mixed sediment environments. The broader implication is that environmental assessments for small-scale coastal infrastructure often simplify sediment budgets and overlook the cumulative effects of upstream land use changes. In the BKW case, historical land cover analysis revealed that over half of the natural wetlands upstream were converted to aquaculture by the mid-1990s. This transformation likely increased fine sediment and organic matter fluxes into the coastal zone. Although these upstream changes were not addressed in the initial project design, they directly influenced the formation of muddy-sand beaches and the post-construction sediment regime.

This study highlights a broader methodological gap in environmental impact study for small-scale fishery and coastal infrastructure projects. Simplified sediment budgets and limited post-construction monitoring often fail to capture the full scope of system responses, particularly in mixed-sediment environments. Therefore, a more integrated process-based approach is required. Incorporating fine sediment dynamics, topographic evolution, and upstream land use change are needed to better anticipate unintended consequences. These insights are not limited to the BKW coast, but they are applicable to other small-scale coastal development projects globally where narrow watershed contributions are often undervalued. Incorporating fine sediment dynamics into feasibility studies is critical for preventing negative impact of shoreline response and for promoting long-term sustainability of coastal systems.

### Causes of beach transformation and role of coastal structures

While large-scale infrastructures such as deep-sea ports, harbors, and extensive coastal protection structures are often linked to coastal degradation^59–61^, recent studies highlight that smaller-scale infrastructures, including fishery piers, can also have significant environmental impacts^18,62–64^. In the case of the Khlong Wan coast, previous legal and environmental reports attributed the transformation of a sandy beach into a mudflat solely to the construction of coastal protection structures, especially seawalls and detached breakwaters^30^. However, our findings indicate that this transformation resulted from a complex interaction of multiple factors, including coastal infrastructure, disrupted sediment transport, and upstream land use changes.

Our observations show that even small structures, such as the 400-m fishery pier, can significantly alter sediment transport and coastal morphology. The pier obstructed the natural longshore transport of sand, historically the dominant sediment source for the BKW coastline, dividing it into two distinct sub-systems: the Wa Ko coastline (updrift) and the Khlong Wan coastline (downdrift) (Figs. 2b and 3a). This disruption led to substantial sand accumulation updrift, while the downdrift coastline experienced sediment deficits.

The 700-m offshore breakwater further influenced sediment dynamics. Its boomerang-shaped design (Fig. 1c) reduced wave energy and current velocities on the lee side during the southwest and transitional monsoons, promoting sand deposition updrift while trapping muddy sediment transported southward during the northeast monsoon. Additionally, an unexpectedly high input of muddy sediment from the Khlong Wan channel, an aspect not considered in the feasibility study, was effectively trapped behind the offshore breakwater, leading to extensive mud accumulation downdrift (Fig. 1b).

Detached breakwaters contributed to sediment deposition by attenuating wave energy. Our observations (Fig. 4a and c) show that these structures caused the seabed to become approximately 1 m shallower than pre-construction levels, with deposited sediment primarily composed of mud. This highlights their role in creating low-energy environments that facilitate fine sediment accumulation, leading to the gradual formation of mudflats above the low tide level along the Khlong Wan coast. The decline in longshore sand transport, coupled with increased fluvial sediment inputs, further contributed to the shift in beach characteristics.

Seawalls along the Khlong Wan coast were built to stabilize the shoreline but have effectively fixed its position, preventing natural landward migration. Over time, wave reflection and scouring can lead to the loss of beaches in front of seawalls^58^. Our analysis revealed that over 95% of the Khlong Wan shoreline in 2023 extended seaward compared to 2002, with an average extension of 18 m (Fig. 2; Table 1). This expansion was mainly due to land reclamation projects by local authorities and private landowners. However, our LiDAR data indicate a lowering of the seabed in front of the seawalls, likely due to wave reflection (Fig. 4d). While seawalls can influence seabed elevation, they do not alter the fundamental composition of beach sediment. Even in cases of erosion, sandy beaches remain sandy; seawalls alone cannot transform a sandy beach into a muddy one, as observed at the Khlong Wan coastline.

Local villagers attributed the beach transformation solely to the construction of seawalls and detached breakwaters, citing altered currents and increased wave reflection as primary causes^30^. While these claims align with known impacts of such structures^29,65,66^they do not fully explain the observed changes. Coastal protection structures can modify hydrodynamics and sediment deposition patterns but do not change the chemical or physical composition of sediments.

Our high-accuracy LiDAR surveys and sediment analysis reveal that the beach transformation was not caused by coastal protection structures alone but rather by a combination of sediment transport disruption and upstream land use changes. Specifically, the conversion of natural wetlands into aquaculture ponds, coupled with intensified aquacultural activities upstream, introduced significant amounts of organic matter and fine sediment into the Khlong Wan channel. These changes directly impacted sediment dynamics, leading to the development of muddy beaches along the downdrift Khlong Wan coastline.

### Adaptive management strategies for coastal sustainability

Recent studies have documented significant coastal degradation associated with smaller-scale infrastructures, especially fishery ports, which adversely affect ecosystems^63,64^tourism^61^and shoreline dynamics^18^often leading to conflicts between stakeholders and government agencies^67^. The legal disputes surrounding the transformation of a sandy beach into a mudflat at Khlong Wan reflect broader challenges in coastal governance. While court rulings may resolve procedural issues, the long-term environmental degradation and the community concerns remain unaddressed.

Given the expanding footprint of coastal development and the documented degradation from even small-scale projects, it is imperative that adaptive management strategies be implemented, especially for small-scale coastal projects. Our study underscores the need to integrate scientific findings into practical coastal management that accounts for the complex interactions among engineered structures, natural sediment dynamics, and upstream land use changes. First, comprehensive pre- and post-development monitoring using high-resolution tools such as LiDAR in combination with long-term shoreline and sediment volume analyses, is essential for establishing baseline conditions to accurately track changes in sediment dynamics and beach morphology. In the BKW case, the lack of pre-construction data on riverine sediment inputs contributed to unforeseen beach transformations.

Second, coastal project designs must incorporate riverine sediment dynamics, particularly in estuarine regions, to balance alongshore and riverine sediment transport. The significant role of fine sediment from the Khlong Wan channel, originally overlooked, emphasizes the need for sediment transport modeling integrated with real-world monitoring data. Third, adaptive management policies should be adopted, enabling real-time decision-making based on continuous coastal processes monitoring; this may include modifying breakwater designs or implementing sediment bypass systems to alleviate sediment trapping and mitigate beach transformation. Fourth, ecosystem-based approaches that prioritize wetland conservation and restoration can help stabilize sediment sources and enhance coastal resilience. Incentives for sustainable aquaculture practices upstream may further reduce the discharge of fine sediment and organic matter into coastal zones.

Finally, robust stakeholder engagement and collaborative governance are crucial. By fostering partnerships among local communities, policymakers, and scientific institutions, management strategies can be tailored to address both environmental and socio-economic priorities. Regional sediment management plans that integrate upstream and downstream processes are also essential to minimize conflicts between natural sediment dynamics and coastal development. These recommendations underscore the importance of combining scientific analysis with practical tools and policies for sustainable coastal development. By adopting these strategies, coastal managers can mitigate the unintended coastal degradation of development projects, enhance ecosystem resilience, and promote conservation in line with global sustainable development goals.

## Conclusions

This study demonstrated how small-scale coastal infrastructure, specifically the 400-m fishery pier and associated offshore breakwaters, can significantly influence sediment dynamics and reshape coastal morphology. Our findings showed that the transformation of the downdrift beach into a muddy coastline was not solely caused by the constructed structures but emerged from the combined effects of engineered modifications, disruption of longshore sediment transport, and upstream land use changes, especially the conversion of wetlands to aquaculture ponds. Our investigation provides important insights for coastal management, showing that removal of seawalls and detached breakwaters alone may not restore sandy beaches and could potentially worsen downdrift erosion. These outcomes highlight the need for integrated coastal management practices that address both anthropogenic and natural sediment processes. We recommend the incorporation of high-resolution monitoring (e.g., UAV-based LiDAR), sediment transport modeling, and sediment budget assessments into coastal planning frameworks. By accounting for both riverine and littoral sediment inputs, decision-makers can better anticipate and mitigate unintended shoreline changes.

While there were some limitations in our study (e.g., lack of pre-construction sediment budget data, and subtidal bathymetry) the processes identified at this site should apply to many other rapidly developing coastal regions facing similar challenges. Based on our findings, we suggest that the use of UAV-based LiDAR for ground elevation data is most useful for analyzing muddy coasts with mild beach slopes and wider tidal ranges, exposing much of the seabed at low tide. In contrast, on sandy beaches with steeper slopes and narrower tidal ranges, sediment volume comparisons can best be made by detailed on-site bathymetric measurements.

Our findings reinforce the need for adaptive management strategies that incorporate ecosystem-based approaches, such as wetland conservation and sustainable aquaculture practices, to stabilize sediment sources and maintain coastal resilience. Strengthening stakeholder engagement and governance frameworks is also essential for bridging the gap between science and policy. As coastal development expands, especially in small and medium-sized communities, science-based, site-specific management approaches will be key to ensuring long-term coastal zone sustainability.

## Supplementary Information

Below is the link to the electronic supplementary material.

Supplementary Material 1

## Data Availability

The datasets generated and/or analyzed during the current study are available from the corresponding author on reasonable request.
