# Supplementary material for: Unintended coastal transformation from small-scale infrastructure and land use change
Source: Sci Rep. 2025 Aug 22;15:30854. doi: 10.1038/s41598-025-15377-y (PMC12373812; doi:10.1038/s41598-025-15377-y)
Supplement: Supplementary file 1 — Supplementary Material 1 [file 41598_2025_15377_MOESM1_ESM.pdf]

Unintended Coastal Transformation from Small-Scale Infrastructure and Land Use Change

Warit Charoenlerkthawin[1,2], Chaiyut Charoenphon[3], William C. Burnett[4], Somboon Otarawanna[5], and Butsawan Bidorn[1,2*]

[1]Department of Water Resources Engineering, Chulalongkorn University, Bangkok 10330, Thailand

[2]Center of Excellence in Interdisciplinary Research for Sustainable Development, Faculty of Engineering, Chulalongkorn University, Bangkok 10330, Thailand

[3]Department of Survey Engineering, Chulalongkorn University 10330, Thailand

[4]Department of Earth, Ocean and Atmospheric Science, Florida State University, Tallahassee, FL 32306, USA

[5]National Metal and Materials Technology Center (MTEC), National Science and Technology Development Agency (NSTDA), Pathum Thani 12120, Thailand

---

[*] Corresponding author's email: butsawan.p@chula.ac.th

Supplementary Materials

1. **Shoreline change analysis and uncertainties**
2. **Grain size analysis**
3. **LiDAR observation**
4. **Tables and Figures**

Table S1: Summary of shoreline position uncertainties and the sources of images used in this study.

Table S2: Summary results from the beach material grain size analysis.

Figure S1: Examples of beach materials updrift and downdrift of the fishery pier: (a) Updrift coastline beach material, classified as fine sand; (b) Downdrift beach material, predominantly composed of fine to medium sand mixed with shells and mud.

Figure S2: Example of DTM and profile used in sediment trapping calculations: (a) 1997 DTM, (b) 2023 DTM, and (c) cross-sectional profile along the red dashed line shown in panels (a) and (b), showing the 1997 and 2023 beach profiles, respectively.

Figure S3: Examples of land use within a 150-ha area located 3 km upstream of the Khlong Wan Channel during 1966–2021. Red rectangles highlight agricultural areas, while blue rectangles indicate aquaculture areas. Panels (a)–(d) show the marsh environment in 1966, 1995, 2002, and 2021, respectively.

Figure S4: Mud sediment supplied from the channel was suspended in the sea. The photo was taken on 24 January 2024 by the author.

5. References

1. Shoreline change analysis and uncertainties

To evaluate the impacts of coastal development on the BKW coastline, historical shoreline positions along a 6.5 km stretch from Khlong Wan Channel to Wa Thon Channel (Figure 1a) from 1966 to 2023 were extracted from aerial photographs and satellite imagery. Aerial photos from 1966, 1984, 1992, and 2002 were sourced from the Royal Thai Survey Department (RTSD), while satellite images from 2010 to 2023 were obtained via Maxar Technologies, CNES, and Airbus satellite data, downloaded free through Google Earth Pro software (version 7.3). To align with modern geospatial analysis standards, all images were georeferenced and orthorectified using ArcGIS version 10.5 (Environmental Systems Research Institute, Inc., California, U.S.), effectively adjusting raster data to a map coordinate system (Charoenlerkthawin et al., 2024; Phanomphongphaisarn et al., 2020; Sok et al., 2022). These images were converted to the Universal Transverse Mercator coordinate system with the World Geodetic System 1984 (WGS1984) as the datum.

Features such as the edge of vegetation, roads, and dikes, which are typically used as shoreline proxies for developed coastlines (Phanomphongphaisarn et al., 2020; Toure et al., 2019; Zhang et al., 2014), were used for delineation of BKW's shoreline positions over six time periods. These proxies are particularly effective for defining the sediment characteristics of the study area, providing a consistent baseline for temporal comparisons of shoreline movement.

The change of shoreline before and after various coastal development projects in the study area was conducted using the Digital Shoreline Analysis System (DSAS) version 5.1, an ArcGIS extension (Himmelstoss et al., 2021), which is widely used for shoreline change studies worldwide (Bidorn et al., 2021; Gharnate et al., 2024; Hzami et al., 2021; Quang et al., 2021; Rafi et al., 2024; Siyal et al., 2022). This system facilitated the generation of 261 transects at 25 m intervals, perpendicular to a baseline defined from the Khlong Wan channel (0+000) to the Wa Thon channel (6+000) in Figure 1a. To determine shoreline movement, we utilized the Net Shoreline Movement (NSM) and End Point Rate (EPR) models, which were used to calculate the displacement and rate of shoreline change for each

transect, (Abd-Elhamid et al., 2023; Akhter et al., 2024; Alharbi et al., 2023; Hossen & Sultana, 2023; Nath et al., 2023).

In the evaluation of shoreline changes using remote sensing techniques, multiple error sources can significantly impact the estimation of shoreline change rates, particularly any uncertainties related to shoreline position and rates of change (Awad & El-Sayed, 2021; Charoenlerkthawin et al., 2022; Gibbs et al., 2019). The shoreline position uncertainties (E_{sp}) include shoreline proxy offset (E_{po}), tidal fluctuation (E_{tf}), seasonal variation (E_{sv}), digitizing error (E_{de}), and toposheet survey error (E_{ts}), and it can be estimate using Eq. S1. Since the shoreline proxies we used, includes vegetation lines and structure's edges, are independent of tidal influence, the shoreline position uncertainties are mainly associated with pixel errors of the aerial and satellite imagery (E_{pe}) and rectification errors of the orthorectification or georeferencing processes (E_{re}) (Awad & El-Sayed, 2021; Charoenlerkthawin et al., 2022; Gibbs et al., 2019). As a result, the terms E_{po}, E_{tf}, E_{sv}, E_{de} and E_{ts} are considered negligible (Hapke et al., 2010; Santos et al., 2021; Sok et al., 2022). The uncertainty of the estimated shoreline change rate (U_i) along each transect is typically determined by the quadratic sum of the uncertainties for each year, divided by the number of years from the first to the last shoreline assessment, as shown in Eq. S2 (Charoenlerkthawin et al., 2022; Rajasree et al., 2016; Sok et al., 2022).

$$E_{sp} = \sqrt{E_{po}^2 + E_{tf}^2 + E_{sv}^2 + E_{de}^2 + E_{ts}^2 + E_{pe}^2 + E_{re}^2} \quad \text{(S1)}$$

$$U_i = \frac{\sqrt{U_1^2+U_2^2+\cdots+U_3^2}}{Year_n - Year_1} \quad \text{(S2)}$$

Where, U_i, i=1, 2,…, n, represents the uncertainty for a given transect at shorelines 1, 2,…, *n*; *n* is the total number of shorelines analyzed. $Year_n$ is the year of the most recent shoreline measurement, and $Year_1$ is the year of initial shoreline measurement. The data and errors associated with the orthorectification process are summarized in Table S1.

2. Grain size analysis

Dry Sieve Analysis:

Dry sieve analysis is a fundamental technique in soil engineering used to determine the grain size distribution of coarse, non-cohesive materials such as sand and gravel. The process begins with sample preparation, where the sediment sample is thoroughly dried in an oven at 110°C to eliminate moisture, ensuring accurate weight measurements. Once dried, a representative sample, typically weighing around 500 grams, is placed in the top sieve of a stack of standard sieves arranged in decreasing mesh size. The sieves are then placed in a mechanical sieve shaker, which agitates the stack for a set period, usually between 10 to 15 minutes. This allows the particles to pass through the sieves based on their size. After sieving, the material retained on each sieve is weighed, and the weight is expressed as a percentage of the total sample weight. The results are then used to construct a grain size distribution curve, plotting the cumulative percentage passing versus sieve size. This method is conducted according to ASTM D6913/D6913M-17: "Standard Test Methods for Particle-Size Distribution (Gradation) of Soils Using Sieve Analysis," and is most effective for analyzing materials with particle sizes greater than 0.075 mm, such as sands and gravels.

Wet Sieve Analysis:

Wet sieve analysis is particularly useful for sediment samples containing a mixture of cohesive materials (such as clay) and non-cohesive materials. This method begins similarly to dry sieve analysis, with an additional step to break down cohesive materials. The sample is first soaked in water to break down any agglomerates, ensuring that finer particles are adequately separated from the coarser ones. The sample is then washed through a 0.075 mm (No. 200) sieve to remove finer particles, such as silt and clay, from the coarser fraction. After this washing step, the remaining coarse fraction is dried and subjected to dry sieve analysis as previously described. Both the coarse fraction (retained on the sieve) and the fines (passing through the sieve) are collected, dried, and weighed. The results from both the wet and dry sieving steps are combined to plot the grain size distribution curve. This method is performed according to ASTM D1140-17: "Standard Test Methods for Determining the Amount of Material Finer than 75-µm

(No. 200) Sieve in Soils by Washing." Wet sieve analysis is ideal for soils where fines are present, ensuring accurate separation and classification of different particle sizes.

Hydrometer Analysis:

Hydrometer analysis is employed to determine the grain size distribution of fine-grained soils, such as silts and clays, which are too small to be analyzed effectively using sieves. In this method, the soil sample is first dispersed in a solution of water and a dispersing agent, typically sodium hexametaphosphate, to prevent the fine particles from clumping together (flocculation). The soil suspension is then transferred to a graduated cylinder, and a hydrometer is used to measure the specific gravity of the suspension at various depths and time intervals. These readings allow for the determination of particle settling rates based on Stokes' law. As the particles settle, the hydrometer readings decrease over time, and these readings are recorded at specific intervals, such as 1, 2, 4, 8, 15, 30, 60, and 120 minutes. The recorded data are used to calculate the percentage of particles finer than a given size, which is then plotted as a grain size distribution curve. Hydrometer analysis follows ASTM D7928-17: "Standard Test Method for Particle-Size Distribution (Gradation) of Fine-Grained Soils Using the Sedimentation (Hydrometer) Analysis," and is crucial for soils where the fine fraction is significant, providing detailed insights into the silt and clay content within the sample.

3. LiDAR observation

LiDAR Survey Setup:

For the coastal survey, seabed elevation data were collected along a 3 km stretch of the coastline between Ban Khlong Wan and Ban Wa Ko using a DJI Matrice 300 RTK drone (SZ DJI Technology Co., Ltd., China) equipped with a Zenmuse L1 LiDAR sensor (SZ DJI Technology Co., Ltd., China). This setup is renowned for its precision, offering a maximum point capture rate of 480,000 points per second, which allows for high-resolution topographical mapping (Charoenlerkthawin et al., 2024; Štroner et al., 2021; Štroner et al., 2023). The LiDAR survey provided a dense point cloud with approximately 5 cm spacing between points, ensuring comprehensive coverage of the surveyed area.

Ground Truthing and Accuracy Verification:

To validate the accuracy of the LiDAR data, ground truthing was conducted using a STONEX S10 GNSS device (Viale dell'Industria 53 | 20,037 Paderno Dugnano (MI), Italy), which is recognized for its high precision in benchmarking and checkpoint positioning (Belcore et al., 2022; Kiliszek & Kroszczyński, 2020). Benchmarks were recorded using a static positioning method, and the data were processed through the National CORS Data Center's service, achieving positional uncertainties within a few centimeters (Chinnarat et al., 2021; Royal Thai Survey Department (RTSD), 2017; Saiyut et al., 2022). Additionally, three to eight ground checkpoints were established using Real Time Kinematic (RTK) survey methods to further validate the UAV-LiDAR data. These checkpoints were positioned on stable structures, such as road concrete slabs, to minimize movement and provide a reliable basis for accuracy comparisons.

RMSE Calculation:

The uncertainty of the LiDAR-derived elevation data was rigorously assessed by comparing the LiDAR measurements against elevations obtained from RTK ground surveys (Buffington et al., 2016; Hladik & Alber, 2012). The Root Mean Square Error (RMSE) was calculated using the following equation:

$$Z_{RMSE} = \sqrt{\sum(z_{LiDARi} - z_{RTKi})^2/n} \qquad \text{(S3)}$$

where z_{LiDARi} represents the LiDAR point cloud elevation at the ith checkpoint, z_{RTKi} is the corresponding RTK survey elevation, and *n* is the number of checkpoints. Based on measurements from five checkpoints, the RMSE for the LiDAR data was found to be only 3 cm, indicating a high level of accuracy (Buffington et al., 2016; Hladik & Alber, 2012).

4. Tables and Figures

Table S1 Summary of shoreline position uncertainties and the sources of images used in this study.

Year	Data	Scale	[2]RMSE (m)	Pixel size (m)	Shoreline Position Uncertainty (m)
1966	Aerial photograph	1:50,000	2.51	1.47	2.91
1976	Aerial photograph	1:50,000	2.43	0.41	2.47
2002	†Orthophotograph	1:4,000	<0.1	1.00	1.00
2013	[1]Satellite image	1:700	0.54	0.72	0.91
2015	[1]Satellite image	1:700	0.56	0.75	0.94
2017	[1]Satellite image	1:700	0.54	0.75	0.93
2019	[1]Satellite image	1:700	0.62	0.75	0.97
2021	[1]Satellite image	1:700	0.61	0.72	0.95
2023	[1]Satellite image	1:700	0.51	0.58	0.77

[1]Downloaded in JPG format at no cost from Google Earth Pro software version 7.3.
[2]RMSE = Root Mean Square Error from the georeferencing process.
†An orthophotograph is an aerial photograph that has been geometrically corrected to have a uniform scale, minimizing distortions and ensuring accurate measurements, which contributes to its low uncertainty.

Table S2 Summary results from the beach material grain size analysis.

Parameters	Wa Ko coastline	Khlong Wan coastline
Total number of samples	14	22
Number of fine-sand samples	10 (†71%)	11 (†50%)
Number of medium sand samples	4 (†29%)	10 (†45%)
Number of mud samples	0 (†0%)	1 (†5%)
median grain size (mm) [SD]	0.143–1.616 [0.45]	0.0165–1.508 [0.48]
Number of samples mixed with silt/clay	††2 (†14%)	11 (†50%)
Mud mixing ratio in each sample (%)	21–23	3–91
[average, SD]	[22, 7.97]	[24, 26.1]
Shell mixing sample (%)	50	81.8
Shell mixing ratio in each sample (%)	0–44	0–41
[average, SD]	[6.5, 15]	[10.7, 12.7]

Note: †percentage of total number of samples.
††sample was found only nearby the south of pier.

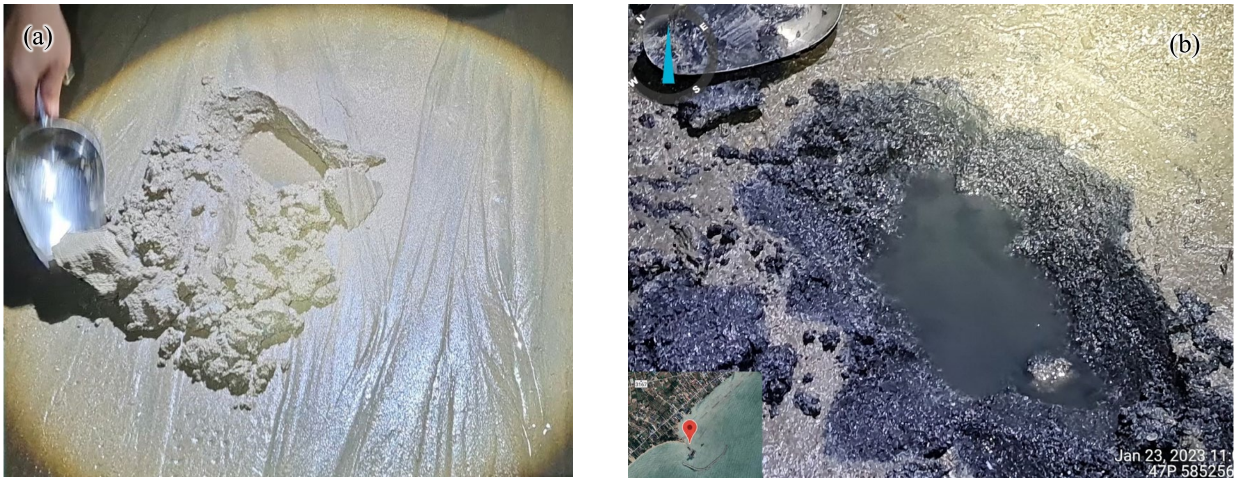

Figure S1 Examples of beach materials updrift and downdrift of the fishery pier: (a) Updrift coastline beach material, classified as fine sand; (b) Downdrift beach material, predominantly composed of fine to medium sand mixed with shells and mud.

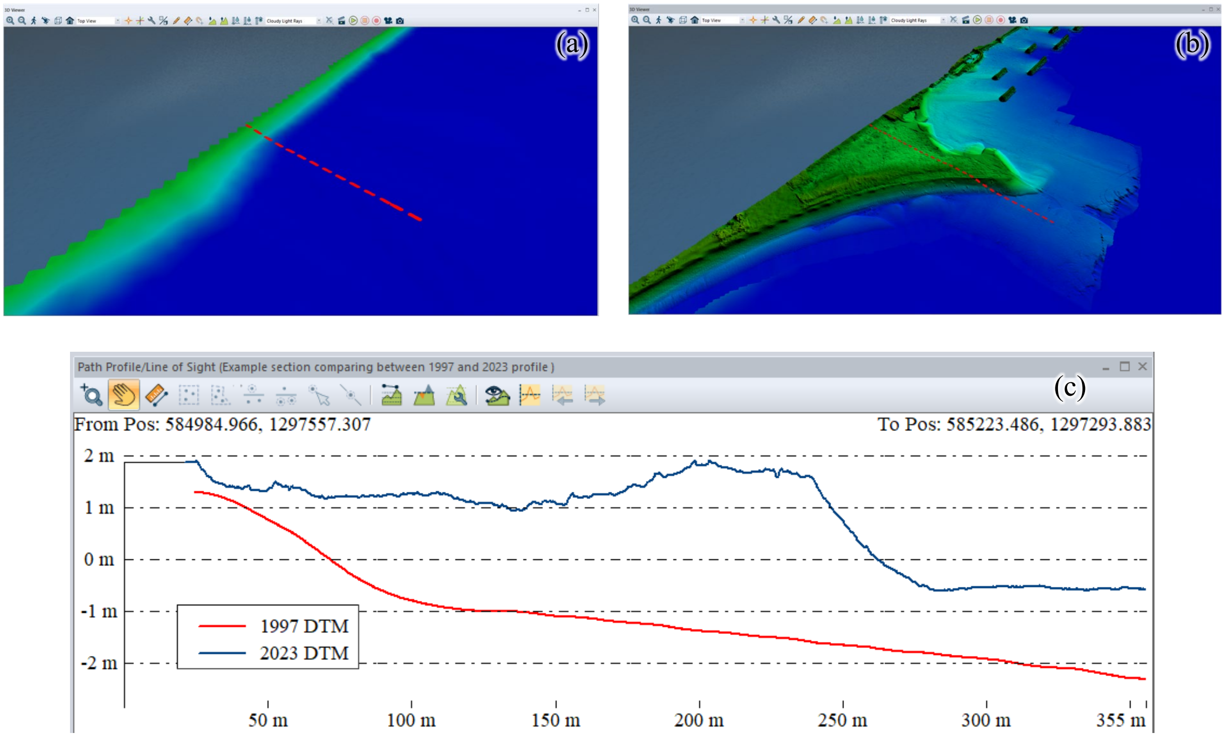

Figure S2 Example of DTM and profile used in sediment trapping calculations: (a) 1997 DTM, (b) 2023 DTM, and (c) cross-sectional profile along the red dashed line shown in panels (a) and (b), showing the 1997 and 2023 beach profiles, respectively.

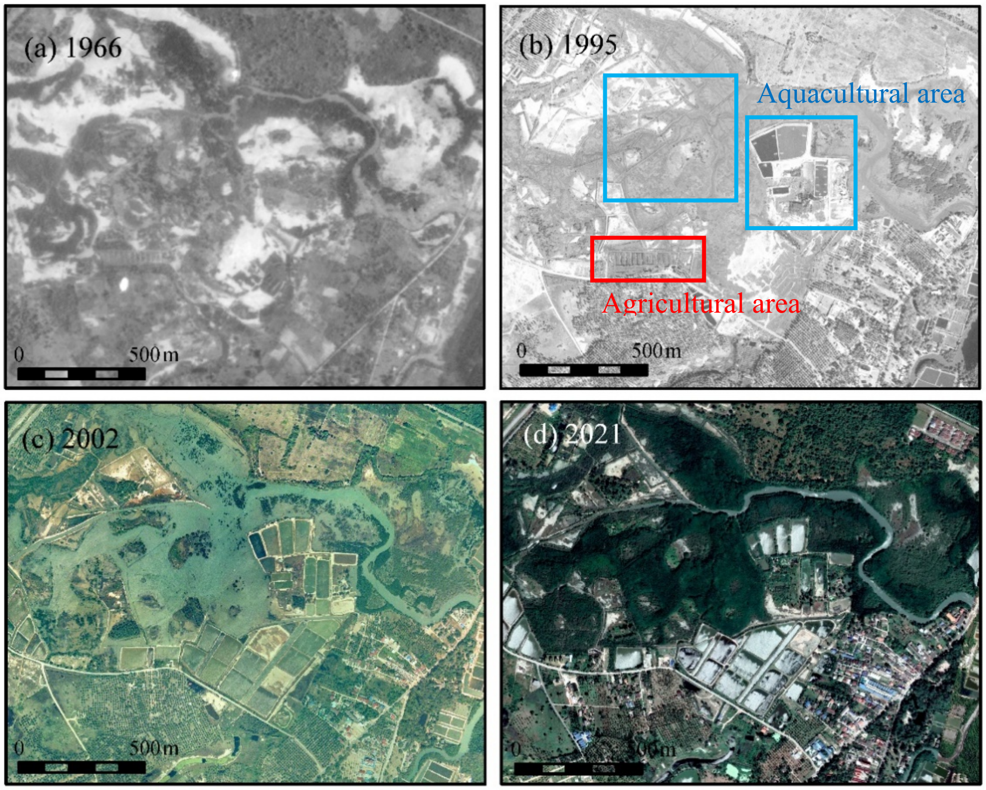

Figure S3 Examples of land use within a 150-ha area located 3 km upstream of the Khlong Wan Channel during 1966–2021. Red rectangles highlight agricultural areas, while blue rectangles indicate aquaculture areas. Panels (a)–(d) show the marsh environment in 1966, 1995, 2002, and 2021, respectively.

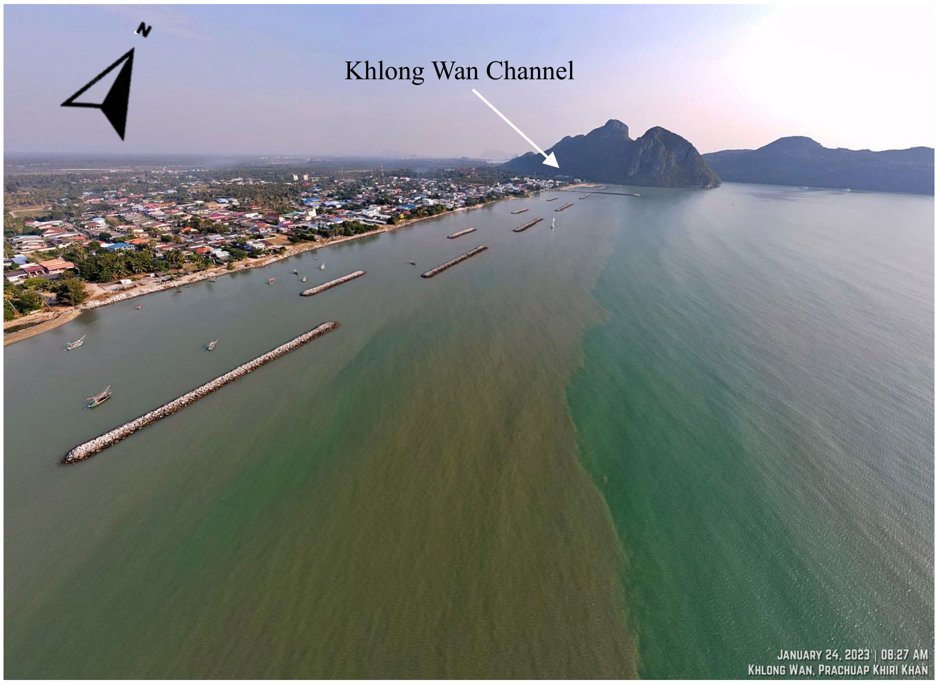

Figure S4 Mud sediment supplied from the channel was suspended in the sea. The photo was taken on 24 January 2023 by the author.

5. References

Abd-Elhamid, H. F., Zeleňáková, M., Barańczuk, J., Gergelova, M. B., & Mahdy, M. (2023). Historical trend analysis and forecasting of shoreline change at the Nile Delta using RS data and GIS with the DSAS tool. *Remote Sensing, 15*(7), 1737.

Akhter, F., Hoque, M. E., & Xu, N. (2024). Geospatial analysis of shoreline and areal dynamics in the Ganges deltaic island of Bangladesh using the GIS-DSAS technique. *Regional Studies in Marine Science, 73*, 103495. https://doi.org/10.1016/j.rsma.2024.103495

Alharbi, O. A., Hasan, S. S., Fahil, A. S., Mannaa, A., Rangel-Buitrago, N., & Alqurashi, A. F. (2023). Shoreline change rate detection applying the DSAS technique on low and medium resolution data: Case study along Ash Shu'aybah-Al Mujayrimah coastal area of the Eastern Red Sea, Saudi Arabia. *Regional Studies in Marine Science, 66*, 103118. https://doi.org/10.1016/j.rsma.2023.103118

ASTM. (2002). Standard test method for particle size analysis of soils (ASTM D422-63). ASTM International.

Awad, M., & El-Sayed, H. M. (2021). The analysis of shoreline change dynamics and future predictions using automated spatial techniques: Case of El-Omayed on the Mediterranean coast of Egypt. *Ocean & Coastal Management, 205*, 105568. https://doi.org/10.1016/j.ocecoaman.2021.105568

Belcore, E., Piras, M., Dabove, P., Massazza, G., & Rosso, M. (2022). Comparison of free and open PPP services for master-base positioning in geodetic disadvantaged areas: Case study along the Sirba River in Sub-Saharan Africa. *Proceedings of GISTAM*.

Bidorn, B., Sok, K., Bidorn, K., & Burnett, W. C. (2021). An analysis of the factors responsible for the shoreline retreat of the Chao Phraya Delta (Thailand). *Science of the Total Environment, 769*, 145253.

Buffington, K. J., Dugger, B. D., Thorne, K. M., & Takekawa, J. Y. (2016). Statistical correction of lidar-derived digital elevation models with multispectral airborne imagery in tidal marshes. *Remote Sensing of Environment, 186*, 616–625. https://doi.org/10.1016/j.rse.2016.09.020

Charoenlerkthawin, W., Bidorn, K., Burnett, W. C., Sasaki, J., Panneerselvam, B., & Bidorn, B. (2022). Effectiveness of grey and green engineered solutions for protecting the low-lying muddy coast of the Chao Phraya Delta, Thailand. *Scientific Reports, 12*(1), 20448.

Charoenlerkthawin, W., Bidorn, K., Burnett, W. C., Sopon, A., Otarawanna, S., & Bidorn, B. (2024). Evaluating the effectiveness of mangrove rehabilitation: A novel approach for sustainable coastal management. *Journal of Environmental Management, 365*, 121673. https://doi.org/10.1016/j.jenvman.2024.121673

Chinnarat, S., Thongtan, T., & Bairaksa, J. (2021). GNSS active control point determinations. *Proceedings of the 18th International Conference on Electrical Engineering/Electronics, Computer, Telecommunications and Information Technology (ECTI-CON), 19–22 May 2021.*

Gharnate, A., Taouali, O., & Mhammdi, N. (2024). Shoreline change assessment of the Moroccan Atlantic coastline using DSAS techniques. *Journal of Coastal Research, 40*(2), 418–435.

Gibbs, A. E., Snyder, A. G., & Richmond, B. M. (2019). *National assessment of shoreline change—Historical shoreline change along the north coast of Alaska, Icy Cape to Cape Prince of Wales (Open-File Report 2019-1146).* U.S. Geological Survey. http://pubs.er.usgs.gov/publication/ofr20191146

Hapke, C. J., Himmelstoss, E. A., Kratzmann, M. G., List, J. H., & Thieler, E. R. (2010). *National assessment of shoreline change: Historical shoreline change along the New England and Mid-Atlantic coasts.* U.S. Geological Survey.

Himmelstoss, E. A., Henderson, R. E., Kratzmann, M. G., & Farris, A. S. (2021). *Digital Shoreline Analysis System (DSAS) version 5.1 user guide (Open-File Report 2021-1091).* U.S. Geological Survey. http://pubs.er.usgs.gov/publication/ofr20211091

Hladik, C., & Alber, M. (2012). Accuracy assessment and correction of a LiDAR-derived salt marsh digital elevation model. *Remote Sensing of Environment, 121*, 224–235. https://doi.org/10.1016/j.rse.2012.01.018

Hossen, M. F., & Sultana, N. (2023). Shoreline change detection using the DSAS technique: Case of Saint Martin Island, Bangladesh. *Remote Sensing Applications: Society and Environment, 30*, 100943.

Hzami, A., Heggy, E., Amrouni, O., Mahé, G., Maanan, M., & Abdeljaouad, S. (2021). Alarming coastal vulnerability of the deltaic and sandy beaches of North Africa. *Scientific Reports, 11*(1), 2320. https://doi.org/10.1038/s41598-020-77926-x

Kiliszek, D., & Kroszczyński, K. (2020). Performance of the precise point positioning method along with the development of GPS, GLONASS, and Galileo systems. *Measurement, 164*, 108009. https://doi.org/10.1016/j.measurement.2020.108009

Nath, A., Koley, B., Saraswati, S., Choudhury, T., Um, J.-S., & Ray, B. C. (2023). Geospatial analysis of short-term shoreline change behavior between Subarnarekha and Rasulpur estuary, east coast of India using intelligent techniques (DSAS). *GeoJournal, 88*(Suppl 1), 255–275.

Phanomphongphaisarn, N., Rukvichai, C., & Bidorn, B. (2020). Impacts of long jetties construction on shoreline change at the western coast of the Gulf of Thailand. *Engineering Journal, 24*(4), 1–17.

Quang, D. N., Ngan, V. H., Tam, H. S., Viet, N. T., Tinh, N. X., & Tanaka, H. (2021). Long-term shoreline evolution using DSAS technique: A case study of Quang Nam province, Vietnam. *Journal of Marine Science and Engineering, 9*(10), 1124.

Rafi, S., Mourya, N. K., & Balasani, R. (2024). Evaluation of shoreline alteration along the Jagatsinghpur district coast, India (1990–2020) using DSAS. *Ocean and Coastal Management, 253*, 107132.

Rajasree, B., Deo, M., & Nair, L. S. (2016). Effect of climate change on shoreline shifts at a straight and continuous coast. *Estuarine, Coastal, and Shelf Science, 183*, 221–234.

Royal Thai Survey Department (RTSD). (2017). Development of precise geoid model of Thailand (in Thai).

Saiyut, P., Thongtan, T., & Bairaksa, J. (2022). Evaluating the accuracy of web-based maps within automatic web processing services. *Proceedings of the 19th International Conference on Electrical Engineering/Electronics, Computer, Telecommunications and Information Technology (ECTI-CON), 24–27 May 2022.*

Santos, C. A. G., Nascimento, T., Mishra, M., & Silva, R. M. D. (2021). Analysis of long- and short-term shoreline change dynamics: A study case of João Pessoa City in Brazil. *Science of the Total Environment, 769*, 144889. https://doi.org/10.1016/j.scitotenv.2020.144889

Siyal, A. A., Solangi, G. S., Siyal, Z.-u.-A., Siyal, P., Babar, M. M., & Ansari, K. (2022). Shoreline change assessment of Indus Delta using GIS-DSAS and satellite data. *Regional Studies in Marine Science, 53*, 102405. https://doi.org/10.1016/j.rsma.2022.102405

Sok, K., Bidorn, B., Burnett, W. C., Sasaki, J., & Sola, P. (2022). Seven decades of shoreline changes along a muddy mangrove coastline of the Upper Gulf of Thailand. *Earth Surface Processes and Landforms, 47*(6), 1425–1438.

Štroner, M., Urban, R., & Křemen, T. (2023). UAV DTM acquisition in a forested area: Comparison of low-cost photogrammetry (DJI Zenmuse P1) and LiDAR solutions (DJI Zenmuse L1). *European Journal of Remote Sensing, 56*(1), 2179942. https://doi.org/10.1080/22797254.2023.2179942

Štroner, M., Urban, R., & Línková, L. (2021). A new method for UAV LiDAR precision testing used for the evaluation of an affordable DJI Zenmuse L1 scanner. *Remote Sensing, 13*(23), 4811. https://www.mdpi.com/2072-4292/13/23/4811
